# Accuracy Validation of the New Barrett True Axial Length Formula and the Optimized Lens Factor Using Sum-of-Segment Biometry

**DOI:** 10.3390/jcm13164639

**Published:** 2024-08-08

**Authors:** Sumitaka Miyamoto, Kazutaka Kamiya

**Affiliations:** 1 Aira Miyamoto Eye Clinic, Kagoshima 899-5213, Japan; airamiyamotoganka@yahoo.co.jp; 2 Visual Physiology, School of Allied Health Sciences, Kitasato University, Kanagawa 252-0373, Japan

**Keywords:** sum-of-segment biometry, ARGOS, Barrett true axial length formula, optimized lens factor

## Abstract

**Objectives:** This study aims to verify the accuracy of a new calculation formula, Barrett true axial length formula (T-AL), and the optimized lens factor (LF) for predicting postoperative refraction after cataract surgery. **Methods:** We included 156 Japanese patients who underwent cataract surgery using Clareon monofocal intraocular lenses at our clinic between January 2022 and June 2023. Postoperative spherical equivalent was calculated using subjective refraction values obtained 1 month post-surgery. The LFs were optimized so that the mean prediction error (PE) of each calculation formula was zero (zero optimization). We calculated the mean absolute PE (MAE) to assess accuracy and used a Friedman test for statistical comparisons. The accuracy of T-AL and the optimized LFs was compared with that of the conventional Barrett Universal II formula for ARGOS (AR-B) and OA-2000 (OA-B) with equivalent refractive index. **Results:** For T-AL, AR-B, and OA-B, the MAEs ± standard deviations were 0.225 ± 0.179, 0.219 ± 0.168, and 0.242 ± 0.206 D, respectively. The Friedman test showed no statistically significant differences among the three groups. The device-optimized LFs were 2.248–2.289 (T-AL), 2.236–2.246 (AR-B), and 2.07–2.08 (OA-B); the corresponding zero-optimized LFs were 2.262–2.287 (T-AL), 2.287–2.303 (AR-B), and 2.160–2.170 (OA-B). **Conclusion:** There were no significant differences in prediction accuracy among the formulas. However, the accuracy of LF optimization varied by device, with T-AL being closest to the value under zero optimization. This suggests that T-AL is clinically useful for predicting an accurate postoperative refraction without zero optimization.

## 1. Introduction

ARGOS, an innovative swept-source optical coherence tomography (SS-OCT) device (Alcon Laboratories, Geneva, Switzerland), uses sum-of-segment and segmented refractive indices to measure the central corneal thickness, anterior chamber depth (ACD), lens thickness (LT) and vitreous, which is considered to give more accurate axial length (AL) measurements, particularly in long or short eyes [1,2,3,4]. For ophthalmic diagnostics and surgical planning, precisely measuring the AL is key, particularly for procedures involving the implantation of intraocular lenses (IOLs). Unlike devices, such as the OA-2000, which uses a refractive index of 1.3375, ARGOS, which uses a unique refractive index of 1.336 for ACD, yields slightly longer ACD measurements. This disparity underscores the importance of investigating its impact on clinical practice [5,6,7,8,9].

The Barrett Universal II formula (B-UII) [10] uses ACD to predict postoperative refraction. ARGOS version 1.1 was released in 2018, but ARGOS ACD measurements were used as they were with the conventional B-UII. Therefore, the results for the optimized lens factor (LF) of the conventional B-UII, as predicted by ARGOS ACD measurements, may differ from those obtained using other devices. Even if ARGOS accurately measures the AL, the expected prediction accuracy may not be obtained without LF optimization. Amid such concerns, the Barrett true axial length formula (T-AL), an ARGOS-specific calculation formula, was first officially released in Japan ahead of other countries in 2023 via ARGOS version 1.5. The release of T-AL has resolved concerns regarding the accuracy of the conventional B-UII calculated using ARGOS ACD measurements and potentially improved predictive accuracy.

Therefore, this study aimed to provide insights into the optimal selection of calculation formulas for cataract surgery planning by clarifying the effectiveness of T-AL and comparing it with that of the conventional B-UII for ARGOS (AR-B) and OA-2000 (Tomey, Nagoya, Japan) with an equivalent refractive index (OA-B). Furthermore, measurements obtained using ARGOS are expected to influence not only the postoperative refraction predicted by the B-UII, but also the optimized LF. In this study, we also compared and verified changes in the LF during optimization for both ARGOS and OA-2000. To our knowledge, although several studies have examined the predictive accuracy of ARGOS [1,2,3,4,5,6,7,8,9], including T-AL [1,2,3], across various calculation formulas, this is the first report to mention differences in optimized IOL individual constants for the same formula between devices. This highlights the need to consider both device- and formula-specific factors in cataract surgery planning.

## 2. Materials and Methods

### 2.1. Device Protocol

This study used ARGOS version 1.6.0, which utilizes SS-OCT with a wavelength of 1060 nm and a 20 nm bandwidth to record two-dimensional optical coherence tomography (OCT) data of the full eye. The different biometry parameters, from the corneal surface to the retinal pigment epithelium, are measured with the OCT, considering different refractive indices: 1.376 for the cornea, 1.336 for the aqueous (ACD) and vitreous fluids, and 1.410 for the lens. The AL for ARGOS is calculated as the sum of these parameters. Keratometry (K) is obtained from OCT images using a 2.2 mm diameter ring comprising 16 LEDs, with a corneal refractive index of 1.3375 applied for a precise corneal curvature measurement [1,2,3,4,5,6,7,8,9].

Meanwhile, OA-2000 version 4H1/7P, which was used in this study, is based on Fourier-domain technology and utilizes SS-OCT with a variable-wavelength laser at 1060 nm to perform 41 horizontal A- and V-scans. All parameters, including ACD, LT, and vitreous, are measured with the B-scanning mode using an equivalent refractive index of 1.3375. K values were measured using the Placido disc-based topography technique by projecting nine rings, each with 256 points, onto a 5.5 mm zone of the cornea, obtaining diameters of 2.0, 2.5, and 3 mm with a refractive index of 1.3375 [5,11,12]. For this study, a default 2.5 mm diameter was used.

### 2.2. Study Design

This single-center, retrospective, observational study included patients who underwent cataract surgery with the implantation of a Clareon (Alcon) monofocal (CNA0T0) and Clareon Toric (CNW0Tx) IOL at our institution between January 2022 and June 2023 (201 cases, 356 eyes).

This study was approved by the Ethics Committee of Aira Miyamoto Eye Clinic on 10 July 2023 (approval ID: Airi2023-1). All patients received a detailed explanation of the surgical procedure by the surgeon, and written consent was obtained from the patients for the use of surgical videos, preoperative and postoperative examination results, and necessary research information, such as age and sex, for academic presentations and study purposes. All patient data were anonymized, and personal information was protected. Patients were provided with the opportunity to decline participation.

### 2.3. Setting

The LF and A constants for Clareon published by the manufacturer were 1.936 and 119.1, respectively. The LFs of the B-UII are generally derived from the A constant of the SRK/T formula [13]; hence, we examined both the LFs and A constants for each formula. OA-B was calculated using measurements from OA-2000, whereas AR-B and T-AL were calculated using measurements from ARGOS. First, the A constants and LFs of OA-B, AR-B, and T-AL were optimized for the surgeon by each device and formula (device optimization) based on cases from the study period and from earlier where Clareon IOL was implanted. The A constants and LFs were optimized using the built-in software in each device. For OA-2000, optimized A constants were converted to LFs. However, the method for calculating LFs in ARGOS was not disclosed. Next, in the case of this study, after calculating the prediction error both before and after device optimization, the LF and A constants were further optimized so that the mean prediction error from each calculation formula was zero (zero optimization) using Excel’s (Microsoft, Redmond, WA, USA) Data/What-If Analysis/Goal Seek function, according to guidelines published by Hoffer et al. [14]. Subsequently, the mean absolute prediction error (MAE) of each formula was calculated. Each eye in this study was measured using ARGOS and OA-2000 on the same day.

### 2.4. Inclusion and Exclusion Criteria

The inclusion criteria were eyes with an upper transconjunctival single-plane incision from 90°, an incision width of 2.5 mm, and a single side-port incision at 0° made using the Verion Image Guided System (Alcon); eyes with complete intracapsular fixation of the IOL; and eyes with no intraoperative or postoperative complications. One eye of each patient was included in the study. The eye with the better corrected visual acuity (CVA) was included in the study if both eyes met the inclusion criteria. The exclusion criteria were a history of refractive surgery and corneal disease, such as pterygium and corneal endothelial disorders, as well as poor postoperative CVA or the poor reliability of measurements and refraction information owing to personalized optimization by ARGOS.

### 2.5. Primary Outcome Measures

The primary outcome measures for each formula compared to verify the accuracy of T-AL were the LFs and A constants after zero optimization, MAE, median AE (MedAE), the standard error (SE), 95% confidence intervals around the AE, and the rates of cases with the AE within 0.25 and 0.5 D (rates within 0.25 and 0.5 D). All cases underwent these analyzes and subgroup comparisons for CNA0T0 and CNW0Tx. Furthermore, the average K, ACD, and AL of each eye per device were assessed to examine the measurement accuracy of ARGOS and OA-2000.

### 2.6. Retrospective Analysis Methods

Statistical analyzes were performed using Excel (version 2406, Build 16.0.17726.20078, 64-bit) from the Microsoft 365 suite and JAMOVI version 2.3.28.0 (The Jamovi Project). This retrospective study was conducted at a private clinic. The mean postoperative duration and standard deviation (SD) were 31.2 ± 3.8 d. Testing of CVA and subjective refraction values, including spherical and cylindrical components, was conducted at a distance of 5 m by an orthoptist, and the results were converted to CVA (logMAR) and spherical equivalent refraction values. The normality of the AE for each formula was tested using the Shapiro-Wilk test, confirming a non-normal distribution (*p* < 0.05 for all formulas). Non-parametric tests do not assume normality, making them suitable for analyzing skewed data and ensuring reliable comparisons across groups. The Friedman test and Cochran’s Q test were used to compare the MAE and rates within 0.25 and 0.5 D of each formula, respectively. When significant differences were found, the Durbin–Conover method or McNemar test was used for post hoc analysis with Bonferroni correction. The Wilcoxon signed-rank test compared measurements obtained using ARGOS and OA-2000, and the Mann–Whitney U test was used to compare subgroups (CNA0T0 and CNW0Tx).

The sample size was calculated with a margin of error of 0.05 and a 95% confidence interval (G*Power 3.1.9.7), suggesting a minimum of 54 eyes for the CNA0T0 subgroup and 36 eyes for the CNW0Tx subgroup. This calculation was based on preliminary data from the initial 20 eyes of each subgroup during the study period to ensure robust and unbiased results. A post hoc analysis of the entire dataset was conducted with *n* = 156 using two tails and yielded a power of 0.999 and an alpha level of 0.05.

## 3. Results

### 3.1. Participant Characteristics and Measurements

Among the 156 eyes of the 156 Japanese cases included in this study (mean age ± SD: 74.7 ± 7.2 years; male, 55.1%), 80 and 76 eyes underwent CNA0T0 and CNW0Tx implantation, respectively. The measurements obtained using ARGOS and OA-2000 are summarized in Table 1. Significant differences were observed between the ARGOS and OA-2000 measurements for average K, ACD, and AL using the Wilcoxon signed-rank test (*p* < 0.001), indicating a strong positive correlation (r > 0.99). No significant differences were found in preoperative corneal astigmatism measured by both devices using the Wilcoxon signed-rank test (*p* = 0.210).

The overall mean subjective astigmatism of the postoperative refraction value was 0.48 ± 0.42 D, and the overall mean postoperative CVA (logMAR) was –0.046 ± 0.070. Postoperative CVA was better in the CNA0T0 subgroup, but subjective astigmatism was lower in the CNW0Tx subgroup.

### 3.2. Analysis of LFs and A Constants

The LF and A constants after device and zero optimizations for each formula are shown in Table 1. The LF and A constants after zero optimization were significantly different from publicly available values. Significant differences were observed in the A constants between ARGOS and OA-2000, but minimal differences were observed among the formulas for device and zero optimizations, indicating that the device’s built-in software were optimized accurately. Moreover, for LFs, significant differences were found between ARGOS and OA-2000, and differences were also noted between device and zero optimizations for AR-B and OA-B. In all formulas, the MEs were less than 0.001, confirming that the zero optimization was properly performed.

### 3.3. Comparison of Formula Accuracy

**Figure 1:**
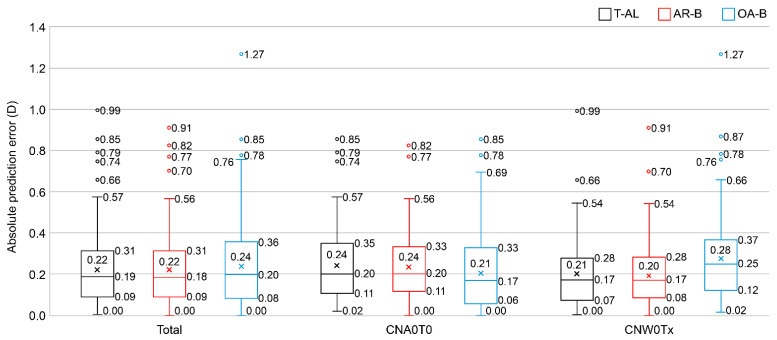
Box plots of the absolute prediction errors for each formula. T-AL: Barrett true axial length formula, AR-B: Barrett Universal II formula for ARGOS, OA-B: Barrett Universal II formula for OA-2000.

Outcome measures are summarized in Table 2 and presented as box plots in Figure 1. Overall, the MAEs and their SDs were 0.225 ± 0.179, 0.219 ± 0.168, and 0.242 ± 0.207 D for T-AL, AR-B, and OA-B, respectively. No significant differences were observed among the three groups (*p* = 0.663). Similarly, the SEs were 0.0144, 0.0135, and 0.0166, and the MedAEs were 0.189, 0.185, and 0.196 D for T-AL, AR-B, and OA-B, respectively. The 95% confidence intervals for the MAEs were 0.197–0.253 D, 0.193–0.246 D, and 0.209–0.274 D for T-AL, AR-B, and OA-B, respectively. In terms of SE, MedAE, and 95% CI, T-AL and AR-B performed slightly better compared to OA-B, but no significant differences were observed. The rates within 0.25 D for T-AL, AR-B, and OA-B were 60.3%, 62.8%, and 59.0%, respectively. No significant differences were found among the three groups using Cochran’s Q test (*p* = 0.622). The rates within 0.5 D for T-AL, AR-B, and OA-B were 92.9%, 95.5%, and 90.4%, respectively. No significant differences were observed among the three groups (*p* = 0.08).

In the subgroup comparisons, almost no significant differences were found between the formulas. The exceptions were the CNA0T0 subgroup, in which the MAE showed a significant difference between AR-B and OA-B using the Durbin–Conover method (after Bonferroni correction, *p* = 0.042), and the CNW0Tx subgroup, in which the rates within 0.25 D showed a significant difference between AR-B and OA-B using the McNemar test (after Bonferroni correction, *p* = 0.003). The rates within 0.5 D were significantly different (*p* = 0.011), but no significant differences were observed using the McNemar test (after Bonferroni correction, *p* = 0.102).

### 3.4. Additional Measurements

**Figure 2:**
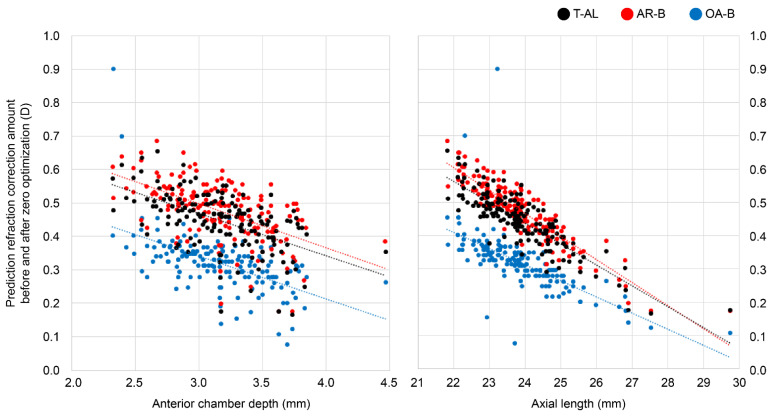
Scatter plots of the prediction refraction correction amount before and after zero optimization vs. anterior chamber depth and axial length. T-AL: Barrett true axial length formula, AR-B: Barrett Universal II formula for ARGOS, OA-B: Barrett Universal II formula for OA-2000.

The publicly available Clareon LF value (1.936) and the LF values after zero optimization for each formula differed significantly. Therefore, we calculated the differences in the predicted postoperative equivalent refraction for each calculation formula before and after zero optimization. The mean absolute differences for T-AL, AR-B, and OA-B were 0.447 ± 0.085, 0.477 ± 0.089, and 0.320 ± 0.085 D, respectively. The differences between T-AL and AR-B, between T-AL and OA-B, and between AR-B and OA-B were statistically significant (*p* < 0.001) when using the Friedman test. Furthermore, evaluating the effect of AL and ACD on zero optimization (Figure 2) revealed that all formulas tended to show larger differences in cases with shorter AL and shallower ACD, with ARGOS showing a slightly larger tendency.

## 4. Discussion

No significant differences in predictive accuracy were observed among the formulas, and the objective of demonstrating the effectiveness of T-AL could not be achieved. The MAE, MedAE, and rates within 0.25 and 0.5 D in this study were 0.219–0.242 D, 0.185–0.196 D, 59.0%–62.8%, and 90.4%–95.5%, respectively. Compared with other reports (0.25–0.42 D, 0.17–0.43 D, 34.1%–56.98%, and 66.0%–89.1%, respectively), all three formulas showed favorable results [1,2,3,4,7,8,9,15,16,17]. Therefore, it is difficult to demonstrate significant effectiveness among these formulas. One possible reason for the favorable results is that, in all cases, the ORA SYSTEM with a VERIFEYE Lynk (Alcon) was used to manage the incision position, the implantation axis of the Toric IOL, and the position and size of the continuous curvilinear capsulorhexis to ensure stable IOL fixation. As a result, the mean postoperative subjective astigmatism was low (0.48 D), minimizing its impact on postoperative spherical equivalent refraction [18,19].

**Figure 3:**
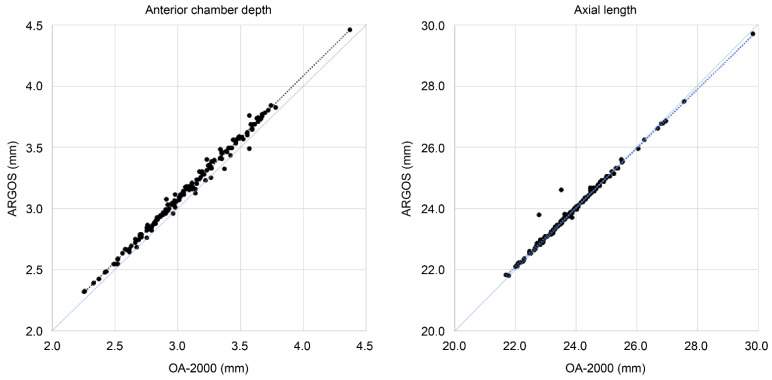
Scatter plots of anterior chamber depth and axial length measured by ARGOS vs. OA-2000.

ARGOS recorded a longer ACD than OA-2000. Moreover, the AL measured by ARGOS was slightly longer in eyes with shorter AL and slightly shorter in eyes with longer AL than OA-2000 (Figure 3). Additionally, mean K measurements showed significant differences between ARGOS and OA-2000, yet a strong positive correlation was observed. These differences are likely due to variations in the measurement methods, such as diameter ring differences (2.2 mm for ARGOS and 2.5 mm for OA-2000). Previous studies have reported similar findings [5,6,7,8,9].

In this study, significant differences were observed between the A constants of ARGOS and OA-2000 after zero optimization. As shown in Table 1, when calculating the IOL power using the SRK/T formula based on the average measurements from each device, a difference of 0.19 D was observed. This difference likely explains why the optimized A constants differed between ARGOS and OA-2000. Although methods for correcting ALs obtained using equivalent refractive indices have been reported [20,21], in this study, larger corrections were required for ARGOS given the small number of short eyes (<22 mm, 2 eyes) and long eyes (>26 mm, 8 eyes), and the favorable outcomes with OA-B. Therefore, we did not consider correcting the AL for OA-2000. Differences in K-values between devices, even with the same AL, can affect prediction refraction. Meanwhile, under zero optimization, when the LFs were converted from the A constants, an OA-B A constant of 119.34 corresponds to an LF of 2.06, whereas achieving an LF of 2.16 requires an A constant of 119.54. Additionally, for a T-AL LF of 2.262, the corresponding A constant would need to be 119.72, which is significantly different from 119.54 for the ARGOS A constant. These results suggest that converting A constants to LF constants is insufficient for accurate predictive calculations using the BU-II formula. These results demonstrate the importance of optimizing the IOL individual constants appropriately for each formula and device.

Focusing on the differences between T-AL and AR-B, T-AL did not respond to changes in predicted results due to ACD measurements for segmented refractive indices but instead improved the accuracy of LF optimization. Therefore, the use of T-AL did not eliminate the need for LF optimization. ARGOS, particularly T-AL, had a smaller difference between device and zero optimization than OA-B, indicating that it may be performing its own optimization. The LF for T-AL and AR-B are nearly identical if LF is optimized for ARGOS. Thus, applying LF derived from AR-B to T-AL may result in minimal alteration in IOL power selection, suggesting that repeating the optimization process for T-AL may not be necessary.

In this study, significant differences were observed between publicly available Clareon constants and those after zero optimization. Significant improvements in the predictive accuracy of Clareon after global optimization in an intraoperative aberrometer were also observed in a previous study [22]. No matter how accurate the calculation formula or device is, if the correct IOL individual constants are not applied, the target refraction cannot be achieved. The differences in the predicted postoperative refraction (0.320–0.477 D) observed before and after zero optimization in the present study indicate that facilities unaware of the LF variations could potentially select incorrect IOL powers relative to the target power. Even if the published A constants are accurate, the actual LF and A constants with ARGOS may differ significantly. ARGOS optimizes the IOL individual constants for all embedded formulas, even when different formulas are used. Therefore, when calculating for a completely new IOL by ARGOS, it is advisable to consider the results for another device until optimization, the trends shown in Figure 2, or both.

The limitations of this study include the small sample size (156 eyes) and short observation period (1 month). Although we were able to standardize the type of IOL and examine the presence or absence of astigmatism correction, we did not have a sufficient number of cases to statistically validate the accuracy for different ALs, as was the case in other studies [1,2,3,4]. Future research should increase the sample size and extend the observation period to approximately 3 months to verify whether the results can be maintained. Additionally, increasing the sample size may allow us to identify the characteristics of T-ALs in patients with short or long ALs, which we failed to demonstrate in this study.

Furthermore, this study only examined two devices, ARGOS and OA-2000, and two formulas, the SRK/T and BU-II. Currently, many other ocular biometry with SS-OCT [11,23] and new generation formulas [15,16] are employed in clinical practice. Expanding the verification of IOL individual constant optimization to include a wider range of formulas and devices could improve the accuracy of postoperative refraction predictions not only for users of ARGOS and OA-2000 but also for a broader population of cataract surgeons, thereby benefiting a larger patient population.

While the predictive accuracy of postoperative refraction has significantly improved, the available IOL powers have largely remained at 0.5-D steps for decades. Even with studies validating excellent predictive accuracy, the limitation of 0.5-D steps constrains target refraction and diminishes clinical significance. To further enhance the precision of refractive cataract surgery, advancements in formulas and devices as well as improvements in IOL manufacturing technology, such as providing 0.25-D steps, will be necessary, and could potentially improve postoperative patient satisfaction.

## 5. Conclusions

T-AL did not respond to changes in predicted results due to ACD measurements for segmented refractive indices but instead improved the accuracy of LF optimization. No significant differences in predictive accuracy were observed among the formulas. However, there were differences in the accuracy of LF optimization depending on the device and formula due to differences in the measurement methods of the parameters or LF optimization methods, T-AL being closest to the value under zero optimization. Therefore, it is recommended to appropriately optimize LF and other IOL individual constants for each device even if the same IOL and formula are used. Future studies should validate the findings of the present study and make efforts to improve the prediction accuracy to further enhance patient satisfaction after cataract surgery.

## Figures and Tables

**Table 1:** Comparative participant characteristics and measurements for all cases and subgroups.

Characteristic	Total (*n* = 156)	CNA0T0 (*n* = 80)	CNX0Tx (*n* = 76)	*p*-Value (Subgroup)
Age (years)	74.7 ± 7.2	73.4 ± 8.2	76.1 ± 5.7	<0.05
Sex (male)	86 (55.1)	35 (43.7)	51 (67.16)	<0.01
ARGOS measurements				
Average K (D)	44.22 ± 1.34	44.43 ± 1.26	44.01 ± 1.39	0.051
Cylinder (D)	1.04 ± 0.65	0.68 ± 0.30	1.43 ± 0.70	<0.001
ACD (mm)	3.17 ± 0.37	3.19 ± 0.37	3.14 ± 0.38	0.394
AL (mm)	23.84 ± 1.16	23.85 ± 1.32	23.83 ± 0.98	0.449
OA-2000 measurements				
Average K (D)	44.10 ± 1.35	44.31 ± 1.29	43.89 ± 1.37	0.068
Cylinder (D)	1.07 ± 0.69	0.66 ± 0.29	1.51 ± 0.72	<0.001
ACD (mm)	3.09 ± 0.36	3.12 ± 0.36	3.06 ± 0.37	0.375
AL (mm)	23.79 ± 1.19	23.81 ± 1.35	23.77 ± 1.01	0.605
Postoperative subjective measures				
Cylinder (D)	0.48 ± 0.42	0.55 ± 0.44	0.42 ± 0.39	<0.05
CVA	–0.046 ± 0.070	–0.055 ± 0.059	–0.036 ± 0.078	<0.001

Values are presented as mean ± standard deviation or *n* (%). Mann–Whitney U test and Cochran’s Q test were used for statistical analyzes. ACD: anterior chamber depth, AL: axial length, CVA: corrected visual acuity (logMAR).

**Table 2:** Comparative analysis of absolute prediction error and optimization parameters for each formula.

Parameter	T-AL	AR-B	OA-B	*p*-Value	P Post Hoc
All cases					
MAE ± SD (D)	0.225 ± 0.179	0.219 ± 0.168	0.242 ± 0.207	0.663	
Standard error	0.0144	0.0135	0.0166		
MedAE (D)	0.189	0.185	0.196		
95% CI (MAE) (D)	0.197–0.253	0.193–0.246	0.209–0.274		
Rate (%)					
<0.25 D	60.3	62.8	59.0	0.622	
<0.5 D	92.9	95.5	90.4	0.08	
CNA0T0 subgroup					
Lens factor					
Device Opt	2.248	2.236	2.08		
Zero Opt	2.262	2.287	2.160		
A constant					
Device Opt	119.52	119.52	119.38		
Zero Opt	119.55	119.55	119.34		
MAE ± SD (D)	0.243 ± 0.186	0.238 ± 0.168	0.209 ± 0.187	<0.05	<0.05 (A vs. O)
Standard error	0.0208	0.0188	0.0209		
MedAE (D)	0.202	0.203	0.167		
95% CI (MAE) (D)	0.202–0.285	0.200–0.275	0.167–0.251		
Rate (%)					
<0.25 D	56.3	56.3	67.5	0.067	
<0.5 D	90.0	95.0	92.5	0.336	
CNW0Tx subgroup					
Lens factor					
Device Opt	2.289	2.246	2.07		
Zero Opt	2.287	2.303	2.171		
A constant					
Device Opt	119.57	119.57	119.37		
Zero Opt	119.61	119.61	119.40		
MAE ± SD (D)	0.206 ± 0.171	0.200 ± 0.167	0.276 ± 0.221	0.174	
Standard error	0.0197	0.0192	0.0254		
MedAE (D)	0.170	0.171	0.247		
95% CI (MAE) (D)	0.166–0.245	0.162–0.238	0.225–0.327		
Rate (%)					
<0.25 D	64.5	73.7	50.0	<0.001	<0.001 (A vs. O)
<0.5 D	96.1	96.1	88.2	<0.05	^1^

The Friedman test and Cochran’s Q test were used for statistical analysis. When significant differences were found, the Durbin–Conover method or McNemar test was used for post hoc analysis with Bonferroni correction. ^1^ No significant differences were found in the post hoc analysis (A and T vs. O, *p* = 0.101 with Bonferroni correction). T-AL: Barrett true axial length formula, AR-B: Barrett Universal II formula for ARGOS, OA-B: Barrett Universal II formula for OA-2000, MAE: mean absolute prediction error, SD: standard deviation, MedAE: median absolute prediction error, CI: confidence interval, Device Opt: device optimization, and Zero Opt: zero optimization.

## Data Availability

The data presented in this study are available on request from the corresponding author. The data are not publicly available to protect the privacy of the patients who participated in this study.
